# Nutritional adequacy in critically ill adults receiving noninvasive ventilation: A descriptive cohort study

**DOI:** 10.1002/jpen.2764

**Published:** 2025-04-23

**Authors:** Francesca Deli, Kevin Whelan, Danielle E. Bear

**Affiliations:** ^1^ Department of Nutritional Sciences King's College London London UK; ^2^ Department of Nutrition & Dietetics Guy's and St Thomas' NHS Foundation Trust London UK; ^3^ Department of Critical Care Guy's and St Thomas' NHS Foundation Trust London UK

**Keywords:** critical care, energy, noninvasive ventilation, nutrition, protein

## Abstract

**Background:**

Noninvasive ventilation (NIV) is increasingly being used in critical care, yet limited evidence exists guiding nutrition practices for patients who are critically ill receiving NIV. This study aimed to describe the nutrition practices and adequacy of nutrition intake among patients who are critically ill receiving NIV.

**Methods:**

This descriptive cohort study included adult patients admitted to critical care who received NIV on ≥3 consecutive days. Prospectively recorded clinical data were retrospectively extracted from electronic medical records and compared between patients who received solely noninvasive ventilation (NIV only) and those who received invasive mechanical ventilation (IMV) and were extubated onto noninvasive ventilation (post‐IMV group).

**Results:**

Of the 220 patients included (107 NIV only; 113 post‐IMV), 142 (64.5%) received exclusive oral nutrition, 66 (30.0%) received artificial nutrition support, and 12 (5.5%) received no nutrition. Enteral nutrition was more prevalent in the post‐IMV group (36 [31.9%] vs NIV only 19 [17.8%]; *P* = 0.01), whereas exclusive oral nutrition was more prevalent in the NIV‐only group (86 [80.4%] vs post‐IMV 66 [58.4%]; *P* < 0.001). Most patients who received purely exclusive oral nutrition (*n* = 152) had inadequate intake (94 [61.8%]).

**Conclusion:**

Most patients with critically illness receiving NIV received exclusive oral nutrition, which was found to be inadequate in the majority. Patients receiving NIV represent a nutritionally at‐risk population, and future studies are needed to understand the barriers to oral intake and the feasibility, safety, and effectiveness of enteral nutrition.

## INTRODUCTION

Patients admitted to critical care frequently require respiratory support either in the form of invasive mechanical ventilation (IMV) provided through an endotracheal or tracheostomy tube or noninvasive ventilation (NIV) delivered through an external interface (eg, nasal, oronasal, face mask, mouthpiece, or helmet). In recent years, there has been increased use of NIV in critical illness in patients who can maintain adequate blood oxygen saturation without IMV.^1^, ^2^, ^3^ NIV is associated with reduced risk of pneumonia; shorter durations of ventilation, critical care stay, and hospital stay; and reduced mortality compared with IMV.^3^

Despite the widespread use of NIV, limited evidence exists on the nutrition practices for this patient group and current nutrition guidelines for critical illness mainly target patients receiving IMV.^4^, ^5^ A recent scoping review of nutrition practices during NIV found that available evidence was limited to mostly small (<100 patients), single‐center observational studies, with reporting mainly focusing on the route of nutrition support with few reporting outcomes related to nutrition interventions.^6^

NIV can represent a barrier to nutrition intake because of risk of aspiration, fasting for intubation, air leaks, and aerophagia, which can affect both oral and enteral intake.^7^, ^8^ In particular, oral intake has been associated with poorer nutrition intake compared with enteral nutrition and parenteral nutrition in patients receiving NIV.^9^ This is often related to the duration of NIV because patients on longer regimens are often unable to tolerate enough time off NIV at mealtimes to allow adequate food intake without experiencing breathlessness. A recent cross‐sectional survey regarding the perspectives of medical and nursing staff on nutrition management during NIV revealed that most clinicians considered nutrition during NIV to be “important or very important.”^8^ However, the lack of evidence‐based guidelines was seen as a significant barrier, resulting in healthcare professionals being reluctant to commence oral or enteral nutrition. This indicates a need to investigate the nutrition practices and barriers to nutrition intake in this population group to guide the development of appropriate nutrition protocols to improve nutrition care during NIV. It is also possible that patients who transition to NIV from IMV may receive different nutrition care, especially as enteral feeding tubes may already be in situ thus overcoming the perceived barrier to insertion while on NIV. However, this has not yet been explored in the literature.

Therefore, the aims of this study were to (1) describe current nutrition practices in critically ill patients receiving NIV; (2) quantify the adequacy of nutrition intake during NIV; and (3) identify the differences in nutrition practices and adequacy between patients who receive NIV only and those who receive NIV postextubation from IMV.

## METHODS

### Study design and participants

This single‐center descriptive cohort study was conducted at Guy's and St Thomas' NHS Foundation Trust, London, UK and approved by the local audit committee (ID 14476) with the need for informed consent waived. The study is reported in accordance with the STROBE guidelines.^10^

Patients were screened for eligibility from a local database of prospectively collected data. Patients were eligible if they were adult (age ≥18 years), admitted to critical care unit (intensive care unit or high‐dependency unit) between December 1, 2021, and August 22, 2022, and required NIV on ≥3 consecutive days. For the purpose of this study, NIV is defined as mechanical ventilation without an artificial airway and includes mask, helmet, and nasal delivery.^11^ Patients receiving NIV for chronic conditions (eg, previous nocturnal NIV for chronic obstructive pulmonary disease), palliative care or long‐term NIV treatment (ie, patients admitted to the chronic respiratory failure unit) were excluded. If patients had multiple critical care admissions during the study period, only the first was considered.

### Data collection

Prospectively recorded routine clinical data were retrospectively extracted from electronic medical records. Baseline characteristics were collected at the point of critical care admission and included age, sex, ethnicity, number of comorbidities, hospital length of stay prior to critical care admission, admission reason, Acute Physiology and Chronic Health Evaluation (APACHE) II score, Sequential Organ Failure Assessment (SOFA) score, body mass index and degree of malnutrition. Global Leadership Initiative on Malnutrition (GLIM) criteria were used to estimate the degree of malnutrition retrospectively based upon weight loss, body mass index, food intake, and inflammation status recorded at admission.^12^

Clinical information was extracted for the duration of critical care admission. The number of episodes of NIV and IMV was recorded for each patient. Cessation of NIV >24 h was considered as the end of that NIV episode. If a patient required multiple NIV episodes, only the first episode (≥3 days) was selected to ensure consistent comparisons between patients. However, if a patient received IMV, only the NIV episode postextubation (≥3 days) was analyzed to facilitate postextubation NIV vs NIV‐only comparisons. For the selected NIV episode, data were collected on the type (nasal high flow or mask delivered) and duration of NIV used and the duration of IMV prior to NIV.

Nutrition information was extracted for all patients for the duration of the NIV episode and included time from NIV to commencement of nutrition, number of days and reasons for nil by mouth, route of nutrition (oral/enteral nutrition/parenteral nutrition), prescription and delivery of enteral and/or parenteral nutrition and oral nutrition supplements. For those patients who received IMV before NIV (post‐IMV group), data on prescription and delivery of enteral and/or parenteral nutrition during IMV were also extracted for comparison.

Additional nutrition information included time from critical care admission to initial dietetic assessment and the estimated energy and protein targets. For patients receiving enteral and/or parenteral nutrition, nutrition adequacy was assessed by calculating energy and protein balances during the episode of NIV. These were determined by comparing the percentages of energy and protein provided with the targets prescribed by dietitians and reported as “energy from all sources” (enteral, parenteral and nonnutrition sources such as intravenous glucose and propofol) or “energy from enteral and/or parenteral nutrition” only. The first and last day of enteral and/or parenteral nutrition were excluded from data extraction to avoid inaccuracies resulting from feed escalation or premature cessation on those days. Nutrition balances were also calculated during the episode of IMV for patients who underwent IMV before NIV, with adjustments made to energy and protein targets on the day of extubation to account for the transition to NIV.

The adequacy of nutrition delivery was calculated based upon the averaged percentage achievement of energy and protein requirements over the NIV episode, and categorized as underfeeding (below 80% of requirements during the NIV episode), adequate (between 80% and 110% of requirements), and overfeeding (above 110% of requirements).^13^

For patients who consumed oral intake during NIV, nutritional adequacy was assessed qualitatively from food charts and was considered “adequate” if >50% of meals were consumed or “inadequate” if <50% of meals were consumed. If food charts were incomplete or unavailable, nursing, medical or dietetic notes were reviewed, and any quantitative or qualitative description of food intake was considered in the identification of whether a patient's oral intake was “adequate” or “inadequate.” If no data on food intake was available or was not quantitative (eg, “patient eating and drinking”), nutrition adequacy was defined as “not recorded.”

### Statistical analysis

Our primary outcome was adequacy of artificial nutrition support (enteral and/or parenteral nutrition) defined as meeting between 80% and 110% of nutrition targets during a single episode of NIV. Our secondary outcomes were as follows: (1) nutrition adequacy from oral intake defined qualitatively from food record charts; (2) comparison of adequacy of artificial nutrition support between patients receiving NIV only and those who received IMV before NIV; and (3) comparison of adequacy of oral intake between patients receiving NIV only and those who received IMV before NIV.

Patients were divided into two groups depending on whether they received NIV only (NIV‐only group) or received IMV and were extubated onto NIV (post‐IMV group). Categorical data were compared between groups using χ^2^ test or Fisher exact test (if data failed to meet χ^2^ assumptions) and for continuous data using unpaired *t* test or Mann‐Whitney test (if data were not normally distributed). For paired analysis in the post‐IMV group comparing data in the same patient during IMV and NIV, paired *t* tests or Wilcoxon tests (if data was not normally distributed) were used for continuous data, and the McNemar test was used for categorical data.

Multivariate logistic regression was used to assess potential clinical factors associated with inadequate artificial nutrition support delivery and their association with clinical outcomes, including survival and readmission to hospital and critical care. The independent variables selected were duration of NIV, prior delivery of IMV, SOFA and APACHE II scores, malnutrition status on admission, and underfeeding during NIV.

Continuous variables are reported as mean and standard deviation (SD) or median and interquartile range (IQR), as appropriate based upon data distribution, whereas categorical variables are reported as frequency (*n*) and percent (%). Normality of continuous data distribution was assessed using the Shapiro–Wilks test for sample sizes <50 and Kolmogorov‐Smirnov test for sample sizes >50.

Statistical analysis was performed using SPSS version 29.0 (IBM Corp). Results with two‐sided *P* values < 0.05 were considered statistically significant.

## RESULTS

**Figure 1:**
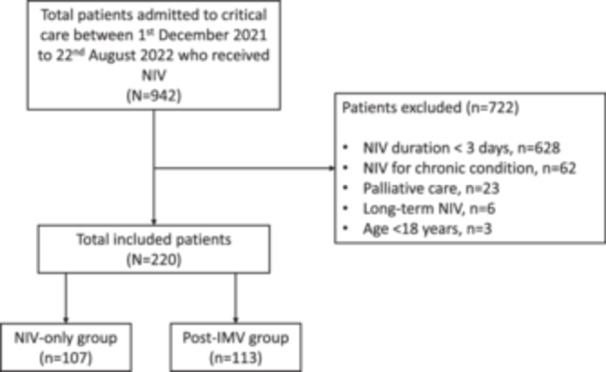
Study flowchart. NIV, noninvasive ventilation; IMV, invasive mechanical ventilation.

A total of 942 patients were screened for eligibility between December 1, 2021, and August 22, 2022 with 722 excluded, mostly because of NIV duration <3 days. In total, 220 patients were included in the final analysis (Figure 1). Nasal delivery was the most common form of NIV delivery (178 [86.4%]) followed by mask. A total of four (1.8%) patients received both mask and nasal delivery and no patients received NIV delivered via a helmet. Of these, 107 received NIV only (NIV‐only group), and 113 were extubated from IMV onto NIV (post‐IMV group).

Baseline characteristics are presented in Table 1 with significant differences seen in age, sex, ethnicity, body mass index, malnutrition diagnosis, illness severity, number of comorbidities, and admission diagnosis between groups. The median number of days of NIV was 3.5 (IQR: 2.8–4.9) days for a total of 85.1 (IQR: 66.0–117.0) h and was similar between groups. However, 52 (24%) patients received NIV for >5 days and 30 (14%) patients for >7 days, and the longest duration of NIV was 19.6 days. Patients in the post‐IMV group received 1 (IQR: 0.5–3.0) day of IMV before NIV.

**Table 1:** Baseline characteristics of critically ill patients receiving noninvasive mechanical ventilation.

	All patients receiving noninvasive ventilation (*n* = 220)^a^	Noninvasive ventilation only (*n* = 107)^a^	Post‐IMV noninvasive ventilation (*n* = 113)^a^	*P* value^b^
Age, median (IQR)	67 (56–74)	68 (57–78)	66 (54.5–72)	**0.05**
Sex, male, *n* (%)	140 (63.6)	56 (52.3)	84 (74.3)	**<0.001**
Admission diagnosis, *n* (%)				**<0.001**
Trauma	5 (2.3)	2 (1.9)	3 (2.7)	—
Postoperative care	83 (37.7)	4 (3.7)	79 (69.9)	—
Respiratory failure	90 (40.9)	74 (69.2)	16 (14.2)	—
Renal failure	10 (4.5)	9 (8.4)	1 (0.9)	—
Circulatory failure	16 (7.3)	7 (6.5)	9 (8.0)	—
Low GCS	3 (1.4)	2 (1.9)	1 (0.9)	—
Multiorgan failure	3 (1.4)	3 (2.8)	0 (0.0)	—
Other	10 (4.5)	6 (5.6)	4 (3.5)	—
BMI	*n* = 211	*n* = 98	*n* = 113	**0.02**
Mean (SD), kg/m^2^	28.6 (6.2)	27.5 (6.9)	29.5 (5.4)	**0.01**
Underweight (<18.5 kg/m^2^), *n* (%)	9 (4.3)	7 (7.1)	2 (1.8)	—
Normal (18.5–24.9 kg/m^2^), *n* (%)	50 (23.7)	31 (31.6)	19 (16.8)	—
Overweight (25–29.9 kg/m^2^), *n* (%)	64 (30.3)	27 (27.6)	37 (32.7)	—
Obese I (30–34.9 kg/m^2^), *n* (%)	53 (25.1)	16 (16.3)	37 (32.7)	—
Obese II (35–39.9 kg/m^2^), *n* (%)	30 (14.2)	14 (14.3)	16 (14.2)	—
Obese III (> 40 kg/m^2^), *n* (%)	5 (2.4)	3 (3.1)	2 (1.8)	—
Number of comorbidities, median (IQR)	3 (2–4)	3 (2–5)	3 (1–4)	**0.02**
APACHE II, median (IQR)	16 (13–21)	17 (14–22)	15 (12–18)	0.00
SOFA, median (IQR)	5 (3–6)	4 (3–6)	6 (4–7)	**<0.001**
GLIM criteria on admission, *n* (%)	*n* = 135	*n* = 71	*n* = 64	**0.01**
No malnutrition	87 (64.4)	37 (52.1)	50 (78.1)	—
Moderate malnutrition	26 (19.3)	18 (25.4)	8 (12.5)	—
Severe malnutrition	22 (16.3)	16 (22.5)	6 (9.4)	—
NIV episodes, median (IQR)	1 (1–1)	1 (1–1)	1 (1–1)	0.41
NIV days, median (IQR)	3.5 (2.8–4.9)	4.0 (2.7–5.5)	3.3 (2.8–4.3)	0.08
NIV hours, median (IQR)	85.1 (66.0–117.0)	95.0 (64.0–132.0)	79.0 (67.1–103.6)	0.08
NIV main type, *n* (%)				0.13
Continuous positive airway pressure	4 (1.8)	4 (3.7)	0 (0.0)	—
High‐flow nasal oxygen	178 (80.9)	85 (79.4)	93 (82.3)	—
High‐flow face mask	22 (10.0)	8 (7.5)	14 (12.4)	—
CPAP and high‐flow nasal oxygen	4 (1.8)	3 (2.8)	1 (0.9)	—
High‐flow nasal oxygen and high‐flow nasal mask	12 (5.5)	7 (6.5)	5 (4.4)	—
IMV episodes, median (IQR)	1 (0–1)	—	1 (1–1)	—
IMV days prior NIV, median (IQR)	0.5 (0.0–1.0)	—	1 (0.5–3.0)	—
Pre‐ICU length of stay, median (IQR)	1 (0–4)	1 (0–4)	1 (1–2)	0.50
Length of ICU stay, median (IQR)	7 (5–13)	7 (5–11)	8 (5–16)	0.10
Post‐ICU length of stay, median (IQR)	8 (4.0–16.0)^c^	12 (4.0–24.5)^d^	5.5 (3.0–12.8)^d^	**<0.001**
Hospital length of stay, median (IQR)	21 (11.0–37.5)^c^	23 (14.0–40.0)^d^	19 (10.0–36.0)^d^	0.10
Hospital survival, *n* (%)	196 (89.1)	89 (83.2)	107 (94.7)	**0.01**
Readmission to ICU within 3 months, *n* (%)	24 (12.2)^e^	15 (17.2)^f^	9 (8.3)^g^	0.08
Readmission to hospital within 3 months, *n* (%)	37 (19.8)^h^	22 (26.5)^i^	15 (14.4)^j^	0.04

*Note*: Bold values indicate statistically significant.

Abbreviations: A&E, accident and emergency; APACHE II, Acute Physiology and Chronic Health Evaluation II; BMI, body mass index; CPAP, Continuous positive airway pressure; GCS, Glasgow Coma Scale; GLIM, Global Leadership Initiative on Malnutrition; HFFM, high‐flow face mask; HFNC, high‐flow nasal cannula; ICU, intensive care unit; IMV, invasive mechanical ventilation; LOS, length of stay; NIV, noninvasive ventilation; SOFA, sequential organ failure assessment.

^a^ The numbers of patients are provided at the top of the column–unless otherwise stated in the row.

^b^ Groups compared using *χ* ^2^ test, Fisher exact test, Mann‐Whitney test, and unpaired *t* test.

^c^ *n* = 200.

^d^ *n* = 100.

^e^ *n* = 196.

^f^ *n* = 87.

^g^ *n* = 109.

^h^ *n* = 187.

^i^ *n* = 83.

^j^ *n* = 104.

The NIV‐only group had a significantly longer postcritical care hospital stay (median: 12 days [IQR: 4–25] vs 6 days [IQR: 3–13]; *P* < 0.001), poorer survival (89 patients (83%) vs 107 patients (95%); *P* = 0.01) and higher rate of 3‐month hospital readmissions (22 patients [27%] vs 9 patients [8%]; *P* = 0.04) compared with the post‐IMV group.

### Nutrition delivery during NIV

Nutrition delivery during NIV is reported in Table 2. There was no difference between groups for the time taken to commence nutrition support (oral/enteral or parenteral nutrition) following initiation of NIV, however, the NIV‐only group took significantly longer to commence artificial nutrition support (enteral and/or parenteral nutrition) compared with the post‐IMV group (16.0 h [IQR: 9.0–33.0] vs 4.5 h [IQR: 0.0–12.8]; *P* = 0.01). During NIV, more patients in the NIV‐only group received oral intake than the post‐IMV group (80 patients [75%] vs 62 patients [55%]), and more patients in the post‐IMV group received enteral nutrition than the NIV‐only group (33 patients [29%] vs 15 patients [14%]; *P* < 0.001).

**Table 2:** Nutrition support delivery during NIV.

	All patients receiving noninvasive ventilation (*n* = 220)	Noninvasive ventilation only (*n* = 107)	Post‐IMV noninvasive ventilation (*n* = 113)	*P* value^a^
Days from NIV start to nutrition commencement,^b^ median (IQR)	0 (0.0–0.1)	0 (0.0–1.1)	0 (0.0–0.1)	0.81
Nutrition started within 24 h of NIV initiation,^b^ *n* (%)	203 (92.3)	94 (97.9)	109 (96.5)	**0.02**
Time from NIV start to EN/PN commencement, median (IQR), h	6.0 (0.5–19.5)	16.0 (9.0–33.0)	4.5 (0.0–12.8)	**0.01**
Main route of nutrition support during NIV, *n* (%)				**<0.001**
NBM	12 (5.5)	9 (8.4)	3 (2.7)	—
Oral	142 (64.5)	80 (74.8)	62 (54.9)	—
Enteral nutrition	48 (21.8)	15 (14.0)	33 (29.2)	—
Parenteral nutrition	17 (7.7)	3 (2.8)	14 (12.4)	—
Enteral and parenteral nutrition	1 (0.5)	0 (0.0)	1 (0.9)	—
Assessed by dietitian, *n* (%)	126 (57.3)	63 (58.9)	63 (55.8)	0.68
Days to initial dietetic assessment, median (IQR)	3 (2–5)^c^	3 (2–5)^d^	3 (2–5)^d^	0.70
EN received during NIV, *n* (%)	55 (25.0)	19 (17.8)	36 (31.9)	**0.02**
EN started prior NIV, *n* (%)	43 (56.6)^e^	4 (14.3)^f^	39 (81.3)^g^	**<0.001**
PN received during NIV, *n* (%)	15 (6.8)	3 (2.8)	12 (10.6)	**0.03**
EN and PN received during NIV, *n* (%)	4 (1.8)	0 (0.0)	4 (3.5)	0.12
Energy target, mean (SD), kcal/day	1866 (305)^c^	1840 (303)^d^	1892 (307)^d^	0.34
Protein target, mean (SD), g/day	89 (18)^c^	84 (19)^d^	94 (17)^d^	**0.00**
Total energy provision during NIV^c^ mean (SD), %	84.0 (20.5)^h^	83.6 (24.6)^i^	84.1 (19.1)^j^	0.93
Total energy adequacy during NIV,^c^ *n* (%)	*n* = 71	*n* = 19	*n* = 52	0.92
Underfeeding	28 (39.4)	7 (36.8)	21 (40.4)	—
Adequate feeding	38 (53.5)	11 (57.9)	27 (51.9)	—
Overfeeding	5 (7.0)	1 (5.3)	4 (7.7)	—
Nutrition energy provision during NIV,^c^ mean (SD), %	82.9 (21.6)^h^	81.8 (27.1)^i^	83.4 (19.6)^j^	0.80
Nutrition energy adequacy during NIV,^c^ *n* (%)	*n* = 71	*n* = 19	*n* = 52	0.91
Underfeeding	29 (40.8)	7 (36.8)	22 (42.3)	—
Adequate feeding	37 (52.1)	11 (57.9)	26 (50.0)	—
Overfeeding	5 (7.0)	1 (5.3)	4 (7.7)	—
Protein provision during NIV (%),^k^ mean (SD)	82.4 (24.6)^h^	85.1 (29.4)^i^	81.4 (22.9)^j^	0.58
Protein adequacy during NIV,^k^ *n* (%)	*n* = 71	*n* = 19	*n* = 52	0.58
Underfeeding	30 (42.3)	8 (42.1)	22 (42.3)	—
Adequate feeding	31 (43.7)	7 (36.8)	24 (46.2)	—
Overfeeding	10 (14.1)	4 (21.1)	6 (11.5)	—

*Note*: Bold values indicate statistically significant.

Abbreviations: EN, enteral nutrition; NBM, nil by mouth; NIV, noninvasive ventilation; PN, parenteral nutrition.

^a^ Groups compared using *χ* ^2^ test, Fisher exact test, and unpaired *t* test.

^b^ All forms of nutrition considered, including oral, enteral nutrition, parenteral nutrition, enteral and parenteral nutrition, nil by mouth.

^c^ *n* = 126.

^d^ *n* = 63.

^e^ *n* = 76.

^f^ *n* = 28.

^g^ *n* = 48.

^h^ *n* = 71.

^i^ *n* = 19.

^j^ *n* = 52.

^k^ Only eligible patients receiving enteral and/or parenteral nutrition are included.

Data for energy and protein delivery was available for the 71 patients (19 NIV‐only group; 52 post‐IMV) that received enteral and/or parenteral nutrition during NIV. The mean (±SD) energy delivered during NIV was 84.0% (±20.5%) of the target from all energy sources. The mean protein delivered during NIV was 82.4% (±24.6%) of target. Overall, energy underfeeding occurred in 28 (39%) patients including all energy sources and 29 (41%) patients including enteral and/or parenteral nutrition only, whereas energy overfeeding occurred in five (7%) patients including all energy sources and enteral and/or parenteral nutrition alone. Protein underfeeding occurred in 30 (42%) patients, whereas protein overfeeding occurred in 10 (14%) patients. Energy and protein delivery and feeding adequacy were similar between groups. Regression analysis showed that, of the a priori defined clinical factors, only SOFA score was associated with greater odds of underfeeding of both energy and protein (Table S1).

Data relating to oral intake during NIV are presented in Table 3. Exclusive oral intake during NIV (86 patients [80%] vs 66 patients [58%]; *P* < 0.001) and reports of “inadequate food intake” (58 [67%] vs 36 [55%]; *P* < 0.001) were more prevalent in the NIV‐only group than in the post‐IMV group. Duration of oral nil by mouth status during NIV was similar between groups (median: 0 days [IQR: 0–1]; *P* = 0.10). However, the main reason for being nil by mouth was different between groups, with the NIV‐only group having a higher prevalence of queries for intubation (8 [24%]) and the post‐IMV group experiencing postextubation dysphagia (5 [11%]) and postoperative protocol (16 [34%]; *P* = 0.005). One week after NIV cessation, a total of 167 (76%) patients received exclusive oral intake. Of these, more patients in the NIV‐only group were reported to have “inadequate intake” compared with the post‐IMV group (29 [34%] vs 13 [16%]; *P* = 0.02).

**Table 3:** Oral intake and delivery of oral nutrition support in patients during NIV.

	All patients receiving noninvasive ventilation (*n* = 220)	Noninvasive ventilation only (*n* = 107)	Post‐IMV noninvasive ventilation (*n* = 113)	*P* value^a^
Exclusive oral intake during NIV, *n* (%)	152 (69.1)	86 (80.4)	66 (58.4)	**<0.001**
Exclusive oral intake adequacy during NIV, *n* (%)	*n* = 152	*n* = 86	*n* = 66	**<0.001**
Inadequate	94 (61.8)	58 (67.4)	36 (54.5)	—
Adequate	45 (29.6)	27 (31.4)	18 (27.3)	—
Not recorded	13 (8.6)	1 (1.2)	12 (18.2)	—
Exclusive oral intake 1 week after NIV cessation, *n* (%)	167 (75.9)	87 (81.3)	80 (70.8)	**0.05**
Exclusive oral intake adequacy 1 week after NIV cessation, *n* (%)	*n* = 166	*n* = 86	*n* = 80	**0.02**
Inadequate	42 (25.3)	29 (33.7)	13 (16.3)	—
Adequate	94 (56.6)	46 (53.5)	48 (60.0)	—
Not recorded	30 (18.1)	11 (12.8)	19 (23.8)	—
ONS prescribed during NIV, *n* (%)	43 (19.5)	29 (27.1)	14 (12.4)	**0.01**
Full ONS prescription delivered during NIV, *n* (%)	*n* = 43 10 (23.3)	*n* = 29 6 (20.7)	*n* = 14 4 (28.6)	0.70
Days NBM orally during NIV, median (IQR)	0 (0–1)	0 (0–1)	0 (0–2)	0.10
Reason for NBM orally during NIV, *n* (%)	*n* = 81	*n* = 34	*n* = 47	**0.01**
Query intubation	12 (14.8)	8 (23.5)	4 (8.5)	—
Procedure/surgery	9 (11.1)	6 (17.6)	3 (6.4)	—
Postextubation dysphagia	5 (6.2)	0 (0.0)	5 (10.6)	—
Awaiting swallow assessment	6 (7.4)	0 (0.0)	6 (12.8)	—
Risk of aspiration	13 (16.0)	6 (17.6)	7 (14.9)	—
Postop	22 (27.2)	6 (17.6)	16 (34.0)	—
Reduced GCS	6 (7.4)	5 (14.7)	1 (2.1)	—
Other	8 (9.9)	3 (8.8)	5 (10.6)	—

*Note*: Bold values indicate statistically significant.

Abbreviations: NIV, noninvasive ventilation; ONS, oral nutrition supplement.

^a^ Groups compared using *χ* ^2^ test and Fisher exact tests.

### Paired comparison of nutrition support during IMV and during NIV

Data relating to adequacy of nutrition support delivery during IMV followed by transition to NIV were collected for the 33 patients in the post‐IMV group that received artificial nutrition support during both of these periods (Table 4). There were no differences for any measures of nutrition support delivery between the IMV and NIV periods, except for percentage protein delivered which was lower during IMV (77.5% [±28.8%]) than during NIV (89.9% [±21.2%]; *P* = 0.01).

**Table 4:** Paired analysis in the post‐IMV group of artificial nutrition support delivery during the IMV and NIV stages.

	During IMV (*n* = 33)	During noninvasive ventilation (*n* = 33)	*P* value^a^
EN days, median (IQR)	4.5 (2–8)	3.5 (3–6.5)	0.91
PN days, median (IQR)	1 (0–8)	1 (0–4)	0.58
Energy received from EN/PN and nonnutritional sources, mean (SD), %	90.3 (27.6)	90.5 (18.7)	0.97
Energy adequacy from EN/PN and nonnutritional sources, *n* (%)			0.31
Underfeeding	9 (27.3)	7 (21.2)	—
Adequate feeding	17 (51.5)	23 (69.7)	—
Overfeeding	7 (21.2)	3 (9.1)	—
Energy received from EN/PN, mean (SD), %	81.4 (27)	89.6 (19.5)	0.090
Energy adequacy from EN/PN, *n* (%)			0.57
Underfeeding	12 (36.4)	8 (24.2)	—
Adequate feeding	18 (54.5)	22 (66.7)	—
Overfeeding	3 (9.1)	3 (9.1)	—
Protein received from EN/PN, mean (SD), %	77.5 (28.8)	89.9 (21.2)	**0.01**
Protein adequacy from EN/PN, *n* (%)			0.19
Underfeeding	14 (42.4)	8 (24.2)	—
Adequate feeding	17 (51.5)	19 (57.6)	—
Overfeeding	2 (6.1)	6 (18.2)	—

Abbreviations: EN, enteral nutrition; PN, parenteral nutrition.

^a^ Paired analysis using McNemar test, paired *t* test, and Wilcoxon test.

### Clinical outcomes

There was no association between nutrition or clinical factors and survival, 3‐month readmission to hospital, or 3‐month readmission to critical care (Table S2).

## DISCUSSION

There are a number of important findings from this study. First, although most patients commenced nutrition support within 24 h of commencing NIV, patients whose sole ventilation source was NIV experienced a delay 3.5 times longer in initiating artificial nutrition support compared with those who were extubated onto NIV. Second, oral intake was found to be the main feeding route during NIV, but higher rates of inadequate oral intake were observed in the NIV‐only group. Third, inadequate energy and protein delivery was seen in approximately 40% of patients receiving artificial nutrition. These results highlight the nutrition‐related vulnerability of this patient group.

In this study, the main route of feeding was oral, followed by enteral nutrition with few patients receiving parenteral nutrition. In contrast, a large study of 1075 patients receiving NIV found that most remained nil by mouth; however, nutrition intake was only examined during the first two days of NIV, compared with the minimum of 3 days in the current study.^14^ Interestingly, differences were found in the main route of nutrition support between the NIV‐only and the post‐IMV group in the current study with more patients in the NIV‐only group receiving exclusive oral intake and more patients in the post‐IMV group receiving enteral nutrition. The high reliance on oral nutrition in this cohort may place patients at high nutrition risk given evidence reporting that patients receiving oral nutrition alone are at risk of energy and protein underfeeding during an admission to intensive care.^15^, ^16^ Indeed, in this study oral nutrition during NIV was mostly considered to be inadequate, more so in the NIV‐only group, although we did not quantitatively measure this. These findings are consistent with others who have observed that 75% of patients receiving NIV consumed <80% of their energy and protein targets.^9^ Although oral and artificial nutrition were not differentiated between, oral intake was associated with poorer nutrition intake.

Interestingly, oral intake in both groups appeared to have improved one week following the cessation of NIV. Others have also reported an improvement in oral intake over time in patients receiving noninvasive respiratory support in an ICU environment.^17^, ^18^, ^19^ However, this improvement of oral intake alongside clinical improvement may also point to NIV hindering oral intake which requires further prospective investigation. Indeed, in a study investigating dietary intake in nonmechanically ventilated adults who are critically ill, including a small proportion receiving NIV, those receiving face or oronasal mask ventilation had lower intakes than those receiving oxygen delivery via nasal cannula (face or oronasal mask: 278 [IQR: 0–1404] kJ; nasal cannula: 836 [IQR: 369–1684] kJ).^17^ The present study did not evaluate the barriers to oral intake and research is needed to comprehensively assess these barriers and develop strategies to overcome them.

The reliance and inadequacy of oral intake in our cohort is particularly concerning since over one fifth of patients were already malnourished upon admission and patients receiving NIV only were often sicker, more malnourished, and less likely to receive artificial nutrition than those who were started IMV first. Interestingly, <40% of patients received oral nutrition supplements during NIV, representing a missed opportunity to improve oral intake. This finding is significant, given the low number of patients in this group who were fed via artificial feeding and may be due to a perception of lower nutrition vulnerability, compared with those receiving IMV. It is also possible that concerns regarding aspiration risk was a contributing factor to the low use of enteral nutrition in this population, as was found in a survey of ICU medical officers and nursing staff, although we did not explore barriers to its use.^8^

The overall use of artificial nutrition support in this study was low, but was two times higher in the post‐IMV group. When artificial nutrition support was used, approximately 40% of patients received inadequate energy and protein provision. This finding is disappointingly high and worse than seen in other critically ill cohorts.^20^, ^21^ There was no difference in underfeeding between patients receiving NIV as the sole method of ventilation and those extubated from IMV, suggesting that IMV delivery before NIV did not affect the ability to deliver nutrition. This challenges the notion that enteral nutrition is contraindicated during NIV because of potential adverse effects such as mask leakage, decreased NIV efficiency and airway complications.^22^ However, reasons for enteral feeding interruptions or association between nutrition route and clinical outcomes were not investigated, and future research is needed to address these knowledge gaps.

The deficit in the provision of enteral and parenteral nutrition and the prevalence of underfeeding may be clinically important, although our regression analysis did not demonstrate associations with survival or 3‐month readmissions to critical care or hospital. Actually, many patients receive NIV for only short periods, indeed we excluded 628 out of 722 patients because of the short duration of NIV, and, therefore, it could be argued that brief periods of underfeeding during short periods of NIV are unlikely to be clinically meaningful. However, some patients receive extended periods of NIV (in the current study 52 [23.6%] patients received NIV for >5 days and 30 [13.6%] patients for >7 days), and, in those patients, underfeeding may be more clinically meaningful. Adequately powered intervention studies should investigate the effect of early artificial nutrition support in this population and on these important clinical outcomes.

### Strengths and limitations

This study has several strengths. First, data were collected for the entire duration of the NIV episode, providing a more comprehensive picture of nutrition practices and delivery during NIV, rather than just at the beginning as other studies have reported.^14^ Second, although one study of nutrition during NIV was conducted in 1075 patients,^14^ this was only for the first 2 days of NIV, whereas the remaining studies were in small samples,^7^, ^17^ and thus the current study is a relatively large population of patients receiving short to medium term NIV.

This study also has some limitations. First, the observational design cannot establish the effect of NIV on nutrition delivery. Second, the retrospective nature of the data collection has led to some missing data. Third, the use of food charts and nursing notes to assess oral nutrition intake may have introduced inaccuracies,^23^ and gold standard methodology, such as weighed food charts and direct observation, should be considered in future prospective studies.

## CONCLUSION

In this study, most patients experiencing critical illness in a single‐center in the United Kingdom receiving NIV relied on oral nutrition which was found to be inadequate. This is concerning, considering the high prevalence of malnutrition on admission, highlighting the nutrition‐related vulnerability of this patient group. Further quantitative and qualitative research is needed to identify potential barriers to oral intake during NIV and explore effective strategies to overcome these including the feasibility and safety of delivering early enteral nutrition and the impact of nutrition support during noninvasive ventilation on clinical outcomes.

## AUTHOR CONTRIBUTIONS

Francesca Deli contributed to methodology, formal analysis, investigation, data curation, the writing of the original draft, and project administration; Kevin Whelan contributed to conceptualization, methodology, resources, review and editing, supervision, and funding acquisition; Daniell E. Bear contributed to conceptualization, methodology, formal analysis, resources, data curation, review and editing, supervision, and project administration.

## CONFLICT OF INTEREST STATEMENT

Francesca Deli declares no conflict of interest. Kevin Whelan has received research grants related to diet and gut health and disease from Almond Board of California, Danone, and International Nut and Dried Fruit Council and has received speaker fees from Danone and Yakult. Kevin Whelan is the holder of a joint patent to use volatile organic compounds as biomarkers in irritable bowel syndrome (PCT/GB2020/051604) for which there is currently no product on the market. In the event of commercialization into a product, the institution and inventor would receive royalties. Kevin Whelan receives royalties from Wiley Publishing in relation to an academic textbook on nutrition support. Danielle E. Bear has received consulting fees from Baxter Healthcare and Nutricia.

## Supporting information

Supplementary material.
