# Supplementary material for: Nutritional adequacy in critically ill adults receiving noninvasive ventilation: A descriptive cohort study
Source: JPEN J Parenter Enteral Nutr. 2025 Apr 23;49(5):609–18. doi: 10.1002/jpen.2764 (PMC12230902; doi:10.1002/jpen.2764)
Supplement: Supplementary file 1 — Supplementary material. [file JPEN-49-609-s001.docx]

**Supplementary material**

**Nutritional adequacy in critically ill adults receiving non-invasive ventilation**

Francesca Deli^1^, Kevin Whelan^1^, Danielle E Bear^1-3^

^1^Department of Nutritional Sciences, King’s College London, London, United Kingdom.

^2^Department of Nutrition & Dietetics, Guy’s and St Thomas’ NHS Foundation Trust, London, United Kingdom.

^3^Department of Critical Care, Guy’s and St Thomas’ NHS Foundation Trust, London, United Kingdom.

**Table S1** – Multivariate logistic regression analysis to determine clinical factors associated with inadequate energy and protein delivery compared to target in patients on NIV receiving enteral nutrition or parenteral nutrition.

|  | Energy and protein underfeeding  (<80% target on average over the NIV episode)  n = 71* | |
| --- | --- | --- |
|  | OR (95% CI) | *p* value |
| NIV duration (days) | 0.693 (0.474-1.013) | 0.058 |
| IMV prior to NIV (yes) | 0.385 (0.041-3.638) | 0.405 |
| APACHE II score | 0.956 (0.835-1.095) | 0.518 |
| SOFA score | 0.694 (0.483-0.998) | **0.049** |
| Malnutrition status (malnourished) | 0.181 (0.025-1.306) | 0.090 |

^*^Only included patients that received artificial nutrition support during NIV. Missing cases: n = 26 due to missing data for GLIM score calculation.

Abbreviations: APACHE II Acute Physiology and Chronic Health Evaluation II; CI confidence interval; IMV invasive mechanical ventilation; NIV non-invasive ventilation; OR odds ratio; SOFA sequential organ failure assessment.

**Table S2** – Multivariate logistic regression analysis to determine nutritional and clinical factors associated with clinical outcomes in 220 patients receiving NIV.

|  | **Survival^a^** | |  | **3-month readmission to hospital^b^** | |  | **3-month readmission to critical care^c^** | |
| --- | --- | --- | --- | --- | --- | --- | --- | --- |
|  | **OR (95% CI)** | ***p* value** |  | **OR (95% CI)** | ***p* value** |  | **OR (95% CI)** | ***p* value** |
| Inadequate energy and protein delivery (underfed) | 1.819 (0.267-12.418) | 0.541 |  | 1.062 (0.187-6.028) | 0.946 |  | 7.684 (0.505-116.942) | 0.142 |
| GLIM criteria (malnourished) | 1.549 (0.167-14.397) | 0.700 |  | 1.177 (0.196-7.065) | 0.859 |  | 2.182 (0.207-22.988) | 0.516 |
| Received IMV (yes) | 11.894 (0.842-168.069) | 0.067 |  | 2.823 (0.284-28.028) | 0.376 |  | 12.156 (0.293-505.084) | 0.189 |
| APACHE II score | 0.980 (0.829-1.160) | 0.817 |  | 1.082 (0.940-1.246) | 0.272 |  | 1.188 (0.997-1.417) | 0.054 |
| SOFA score | 0.906 (0.603-1.361) | 0.634 |  | 0.959 (0.697-1.321) | 0.798 |  | 1.203 (0.824-1.756) | 0.338 |

Missing data: ^a^n = 85, ^b^n = 110, ^c^n = 103 due to patients being transferred to other hospitals or died or data missing for GLIM score calculation.

Abbreviations: APACHE II Acute Physiology and Chronic Health Evaluation II; CI confidence interval; IMV invasive mechanical ventilation; OR odds ratio; SOFA sequential organ failure assessment.
